# Enhanced Thermoelectricity in Metal–[60]Fullerene–Graphene Molecular Junctions

**DOI:** 10.1021/acs.nanolett.3c00014

**Published:** 2023-03-27

**Authors:** Simon A. Svatek, Valentina Sacchetti, Laura Rodríguez-Pérez, Beatriz M. Illescas, Laura Rincón-García, Gabino Rubio-Bollinger, M. Teresa González, Steven Bailey, Colin J. Lambert, Nazario Martín, Nicolás Agraït

**Affiliations:** † Instituto Madrileño de Estudios Avanzados en Nanociencia (IMDEA-Nanociencia), Faraday 9, Ciudad Universitaria de Cantoblanco, 28049 Madrid, Spain; ‡ Departamento de Física de la Materia Condensada, Facultad de Ciencias, Universidad Autónoma de Madrid, C/Francisco Tomás y Valiente 7, 28049 Madrid, Spain; § Organic Chemistry Department, Faculty of Chemistry, Universidad Complutense de Madrid, E-28040 Madrid, Spain; ∥ Condensed Matter Physics Center (IFIMAC) and Instituto Universitario de Ciencia de Materiales “Nicolás Cabrera” (INC), Facultad de Ciencias, Universidad Autónoma de Madrid, C/Francisco Tomás y Valiente 7, 28049 Madrid, Spain; ⊥ Department of Physics, Lancaster University, Lancaster LA1 4YW, United Kingdom

**Keywords:** thermoelectricity, quantum thermopower, fullerenes, molecular electronics, conductive atomic force microscopy

## Abstract

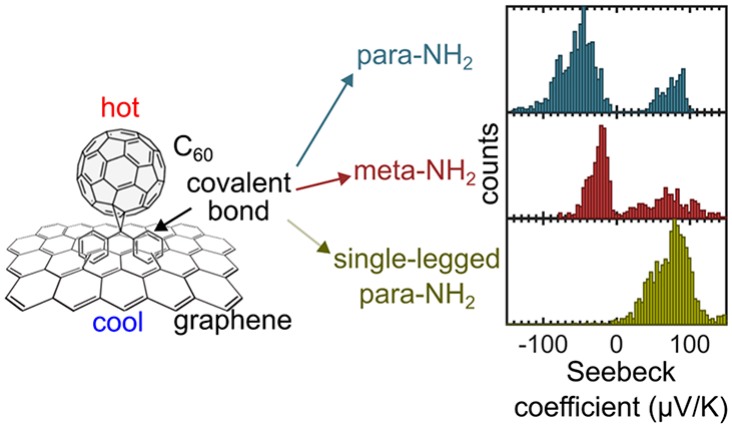

The thermoelectric properties of molecular junctions consisting of a metal Pt electrode contacting [60]fullerene derivatives covalently bound to a graphene electrode have been studied by using a conducting-probe atomic force microscope (c-AFM). The [60]fullerene derivatives are covalently linked to the graphene via two *meta*-connected phenyl rings, two *para*-connected phenyl rings, or a single phenyl ring. We find that the magnitude of the Seebeck coefficient is up to nine times larger than that of Au–C_60_–Pt molecular junctions. Moreover, the sign of the thermopower can be either positive or negative depending on the details of the binding geometry and on the local value of the Fermi energy. Our results demonstrate the potential of using graphene electrodes for controlling and enhancing the thermoelectric properties of molecular junctions and confirm the outstanding performance of [60]fullerene derivatives.

Graphene and [60]fullerene are two Nobel laureated disruptive materials representing new allotropes of carbon whose properties could be fine-tuned upon chemical covalent^1,2^ or supramolecular modification.^3,4^ Therefore, the construction of novel fullerene–graphene architectures could enhance the existing properties of both carbon allotropes and/or bring about new and exciting ones.^5^ In this regard, several [60]fullerene-graphene hybrids have been reported in the references.^6−10^ It is worth noting that, as expected, the covalent wet modification of graphene involves the functionalization on both sides of the graphene layer. However, functionalization of only one side has also been accomplished by using a solid substrate where the graphene layer is deposited.^11−13^ In this sense, in most of the practical applications of graphene, it is actually supported on a solid substrate which could be easily incorporated into an electronic device. Therefore, the chemical functionalization of graphene on a substrate with [60]fullerene may drastically expand the material capabilities by fine-tuning its chemical and physical properties.

Studies of the thermoelectric properties of molecular junctions consisting of organic molecules connected to electrodes provide insight into the charge transport mechanisms at the molecular level.^14^ They also open the possibility of designing new thermoelectric devices with enhanced thermoelectric properties that could find applications in energy management, on-chip cooling, temperature sensing, or energy harvesting.^15−20^ All-carbon hybrid devices show great promise in their ability to exploit desirable thermoelectric properties.^21−25^ One such property is the delicate control of the sign and magnitude of the Seebeck coefficient. This control has been demonstrated, for example, by applying tip pressure to modulate the coupling of the molecule to the electrode^26^ and by tuning intermolecular interactions between C_60_ molecules.^18^

The thermopower of various single fullerene molecules between a gold substrate and a metal tip of Au, Ag, and Pt was studied by Yee et al.^27^ They found that for C_60_, PCBM, and C_70_, the Seebeck coefficient was negative, being largest in magnitude for C_70_ and for the Ag tip (−33 μV/K). These are among the highest measured values for molecular junctions.^14^ Evangeli et al.^18^ found that the thermopower of C_60_ dimers between gold electrodes reaches values of −33 μV/K, almost double that of C_60_ (−18 μV/K). The fact that the thermopower is negative for all these fullerene molecular junctions shows that electron transport takes place through their LUMOs (lowest unoccupied molecular orbitals). In contrast, it has been shown that, in molecular junctions of endohedral fullerenes, the Seebeck coefficient can be either positive or negative depending on the orientation of the encapsulated moiety and also in response to pressure; this behavior was explained by the presence of transmission resonances close to the Fermi level.^17^

Here, we investigate the thermoelectric properties of single-molecule junctions formed by contacting diphenylmethanofullerene molecules (**1**–**3**) bound covalently to graphene on SiO_2_/Si (**GOS-1**, **GOS-2**, and **GOS-3**) as shown in Figure 1a. We find that the Seebeck coefficients of these molecular junctions present typical values of up to 74 μV/K and −56 μV/K, which are much larger in magnitude than the 8.9 μV/K previously reported for single Au–C_60_–Pt junctions. We use density functional theory (DFT) calculations to obtain a detailed understanding of the origin of these values and the molecular configurations responsible for these amazing behaviors.

**Figure 1:**
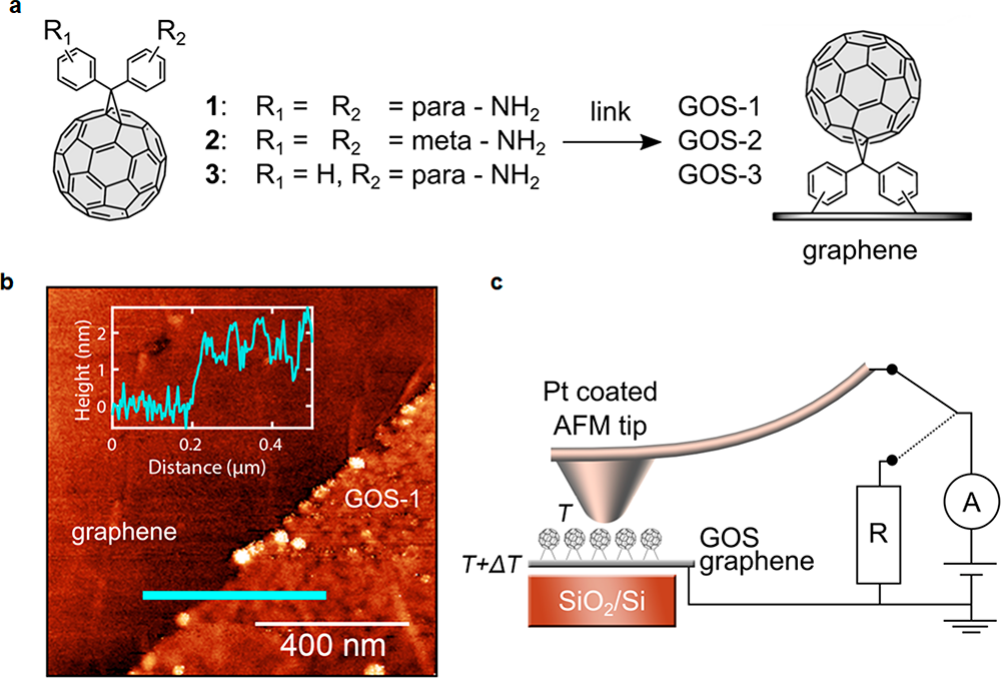
(a) Scheme of the three molecules studied (**1**–**3**) and their covalent link to graphene. (b) AFM topographic image of the edge of an island of **GOS-1** and one profile showing its apparent height. (c) Scheme of the setup for the measurement of the thermoelectric properties.

## Results and Discussion

**Scheme 1:**
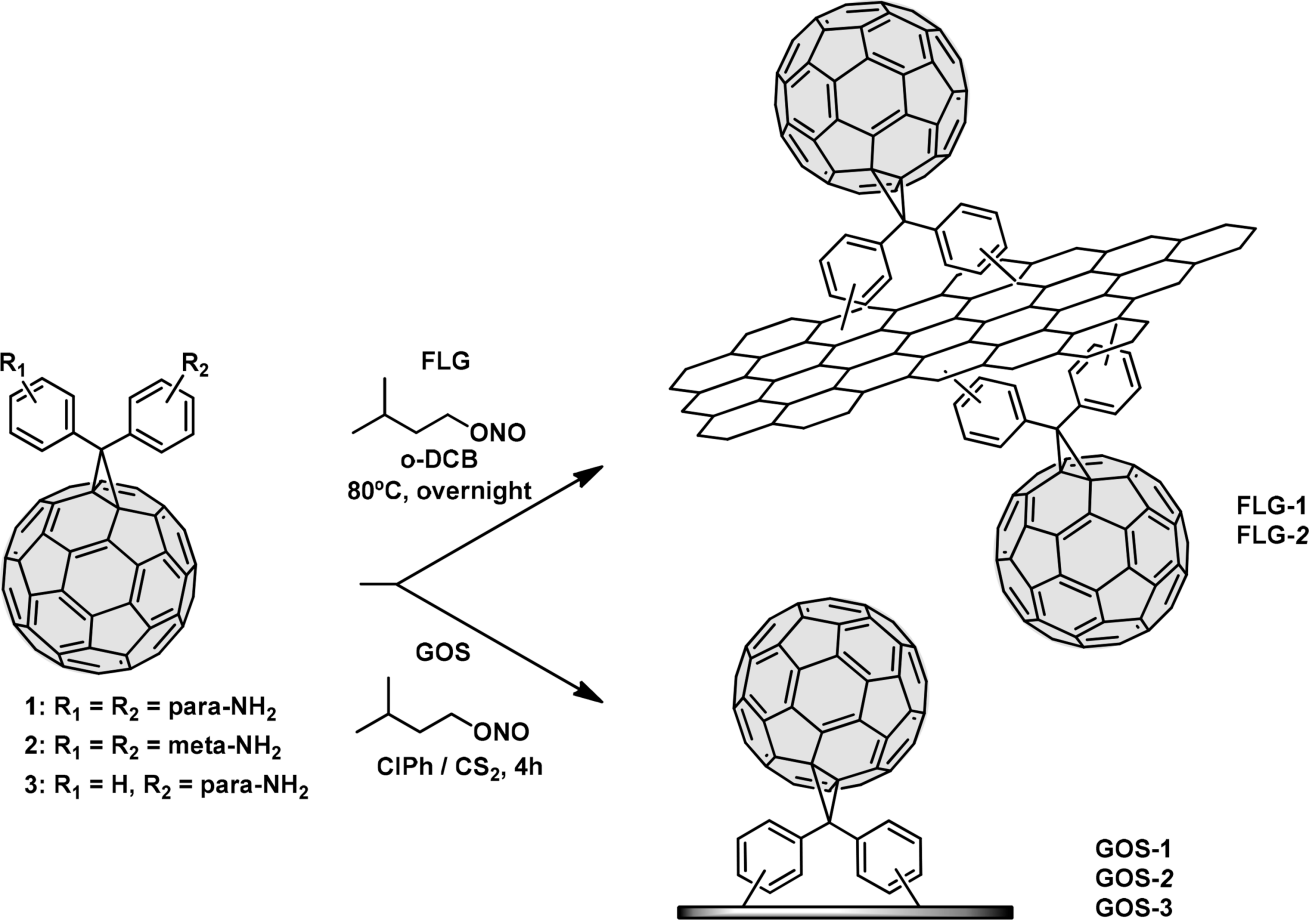
Covalent Functionalization of FLG and GOS through Tour’s Reaction

For the study of the thermoelectric properties, we employed large area monolayer graphene grown by chemical vapor deposition (CVD) on copper foils, cut into smaller pieces and transferred onto SiO_2_. Covalent binding of methanofullerenes **1**–**3** to graphene on substrate (GOS) led to derivatives **GOS-1**, **GOS-2**, and **GOS**-**3** (Scheme 1). The synthesis of compounds **1**–**3** was carried out employing a Bamford–Stevens reaction, which takes place through the *in situ* generation of intermediate diazocompounds with sodium methoxide in the presence of pyridine and [60]fullerene in refluxing *o*-dichlorobenzene (*o*-DCB), affording the final amino or diamino-containing diphenylmethanofullerenes **1**–**3**.^28^ The covalent functionalization of GOS was conducted via aryl diazonium chemistry in the presence of isoamyl nitrite, following a slightly modified protocol of that previously reported by Tour and co-workers.^29^ Thus, the corresponding aryl diazonium salts are generated in advance by adding isoamyl nitrite to a solution of **1**, **2**, or **3** and subsequently drop casted on the GOS. As proof of concept and to validate the covalent functionalization over the graphene grafted on top of SiO_2_/Si, we have also synthesized the analogous all-carbon hybrid materials over few layers graphene (FLG) to achieve a complete characterization of the final derivatives. Pristine FLG was obtained through graphite exfoliation following Colleman’s method with a high degree of purity.^30^ A suspension of FLG in *o*-DCB was immediately reacted with [60]fullerene derivatives **1** and **2** in the presence of isoamyl nitrite providing **FLG-1** and **FLG-2,** respectively (Scheme 1).

**Figure 2:**
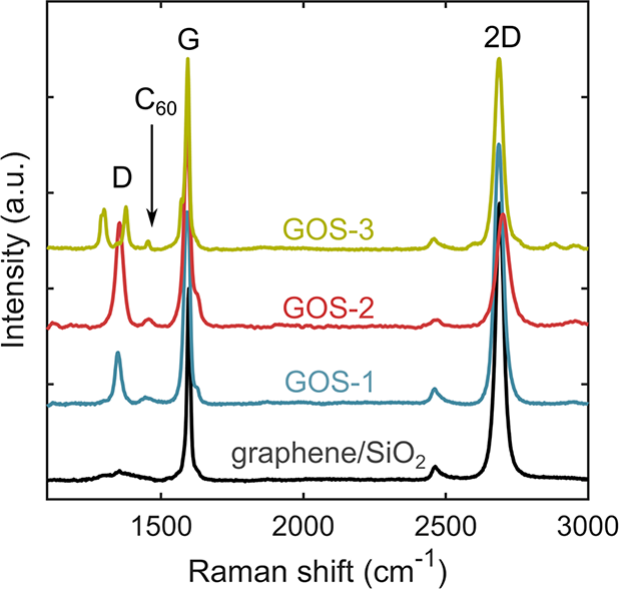
Raman spectra of pristine GOS (black), GOS-1 (blue), GOS-2 (red), and GOS-3 (green) under 532 nm laser excitation wavelength.

Raman spectroscopy under 532 nm laser wavelengths gives insights of the surface functionalization of FLG and GOS. All aggregates present an increase in the D-band with respect to the G-band, which is generally associated with a higher defect degree on the nanomaterials, also attributed to functionalization. This increase is related with a substantial rehybridization from sp^2^ carbon atoms to sp^3^ as a result of the covalent attachment of **1**, **2**, or **3**, respectively. Therefore, the ratio with the G band *I*_D_/*I*_G_ is a way to quantify the covalent functionalization degree of those materials. According to this criterion, in our measurement we found *I*_D_/*I*_G_ ratios of 0.26 for **GOS-1**, 0.54 for **GOS-2**, and 0.23 for **GOS-3** (Figure 2 and Figures S1–S3). Moreover, a new peak around 1457 cm^–1^ is clearly observed for all fullerene aggregates which can be assigned to the pentagonal pinch mode [Ag(2)] of C_60_.^31^ These results indicate a higher functionalization in the case of **GOS-2**, which is also confirmed by the observed *I*_D_/*I*_G_ ratios for **FLG-1** and **FLG-2** (Figure S4). Thus, this evidence confirms the covalent attachment of **1**, **2**, or **3** on the FLG or GOS. A characterization of **FLG-1** and **FLG-2** employing ATG, FTIR, XPS, and TEM has been carried out and is available in the SI.

AFM images of the samples reveal the formation of micrometer-sized molecular islands of **GOS-1**, **GOS-2**, or **GOS**-**3** that partially cover the graphene sheet (see Figure 1b). The measured height is around 2 nm, which is larger than the diameter of a [60]fullerene and consistent with the covalent bonding between the fullerene and graphene.

To characterize the thermoelectric response of the [60]fullerene derivatives on graphene, we used a conductive atomic force microscope (c-AFM) with Pt-coated tips (Multi75-G from BudgetSensors). Figure 1c shows a scheme of the measuring technique, which we have previously described in detail.^18^ In short, the substrate is heated and the tip–substrate temperature difference Δ*T* is determined from direct measurements of the temperatures of the cantilever chip and the substrate. The Pt-coated tip is placed into gentle contact (∼30 nN) with the substrate and held stationary through a feedback loop controller. This allows us to measure current–voltage (*I–V*) characteristics in which the thermoelectric voltage shows as a voltage-offset Δ*V*, from which one can obtain the thermopower or Seebeck coefficient *S* = −Δ*V*/Δ*T*. A zero-calibration of the voltage reading is performed in short time intervals by disconnecting the c-AFM from the *V* source and connecting a resistor *R* instead, facilitating a precise voltage reading. Then we can determine *S* and the conductance *G* (from the slope of the *I*–*V* curves) for a number of given points on the substrate.

**Figure 3:**
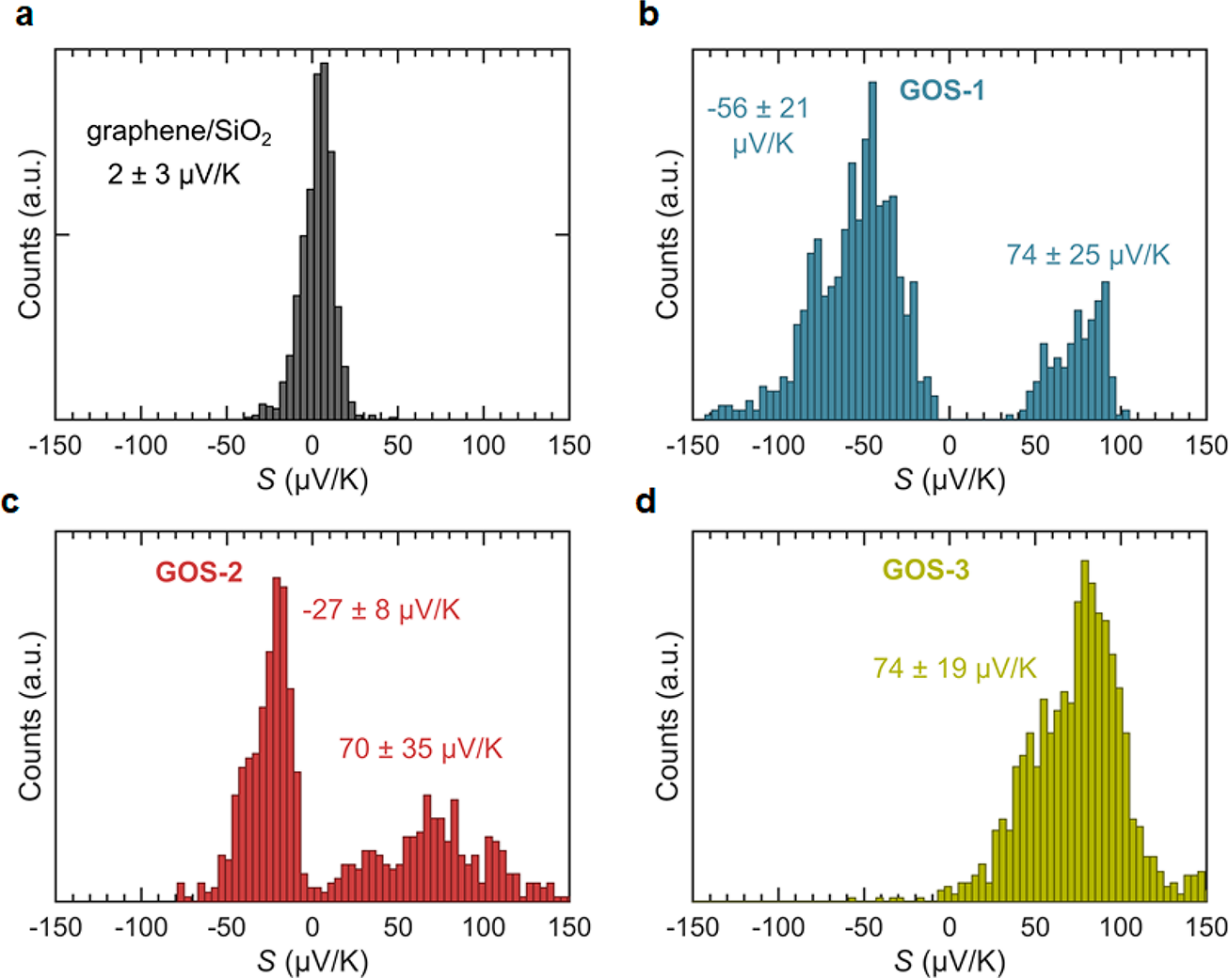
Histograms of the thermopower of junctions formed with (a) graphene, (b) **GOS-1**, (c) **GOS-2**, and (d) **GOS-3**. The uncertainty given for the Seebeck coefficients equals the standard deviation of the distribution of measured values.

Figure 3a shows a histogram of *S* measured with the tip in contact with the graphene. We obtain a very small average value of 2 μV/K, corresponding to the thermopower of the Pt-tip/graphene/SiO_2_ junction. This measurement configuration is totally different to that of previously reported experiments that determine the in-plane thermopower of graphene, which is found to be between −50 and +50 μV/K, depending on doping.^32^

Measurements of the thermopower of **GOS-1** yield two distinct sets of values centered on *S* = −56 μV/K and *S* = 74 μV/K (see Figure 3b). Note that, for a single approach in a given area, only values that correspond to one of the peaks appear, although imaging shows no significant difference between areas of different thermopowers. These Seebeck values are exceptionally high compared with those commonly observed in molecular junctions of other organic molecules.^14^ Interestingly, we find that when the force with which the tip is pushed toward the surface is increased, the tip displaces the molecules, jumps into contact with the graphene, and registers the Seebeck coefficient of graphene, as shown in Figure 3a. Similarly for **GOS-2**, we observe two peaks in the thermopower histogram, centered on *S* = −27 μV/K and *S* = 70 μV/K (see Figure 3c).

The presence of broad distributions with two peak values of the Seebeck coefficient for both **GOS-1** and **GOS-2** suggests the existence of different configurations, in particular the possibility of having molecules bound by the two legs or only by one leg. To test this hypothesis, we prepared samples of **GOS-3**, which can bind with only one leg. Figure 3d shows a histogram of the Seebeck coefficient *S* measured when the tip is in contact with an island of **GOS-3** on graphene. We only find high positive values of *S* centered around *S* = 74 μV/K.

**Figure 4:**
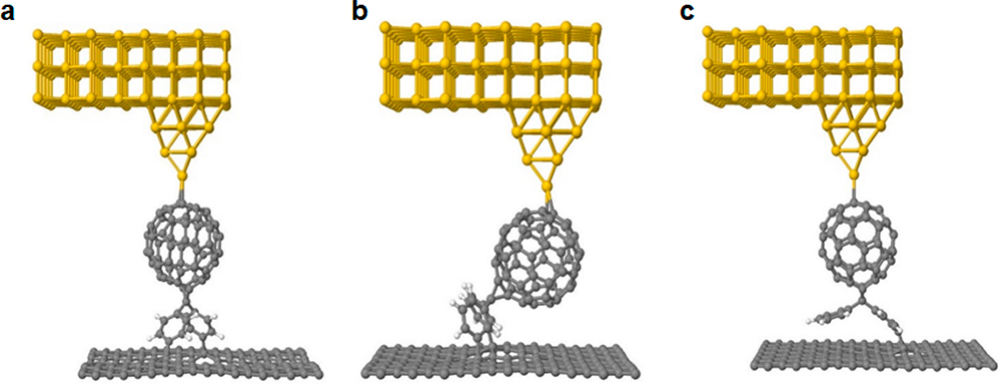
DFT-generated structures of (a) **GOS-1**, (b) **GOS-2**, and (c) **GOS-3** covalently bonded to a graphene sheet. As an archetypal metallic electrode, the top contact (shown in yellow) is chosen to be gold and is attached via a tapered tip to a stable contact point on the C_60_. The processes involved in creating these structures are described in detail in the SI.

To further understand this behavior, we used density functional theory (DFT) combined with quantum transport theory to compute the electrical conductance *G* and Seebeck coefficient *S* of these junctions. Initially, the SIESTA implementation of DFT was used to extract the mean field Hamiltonian of the system for the relaxed geometries shown in Figure 4. The electronic and thermoelectric properties were then calculated using the transport code GOLLUM, starting from the transmission coefficients *T*(*E*), as described in the SI.

**Figure 5:**
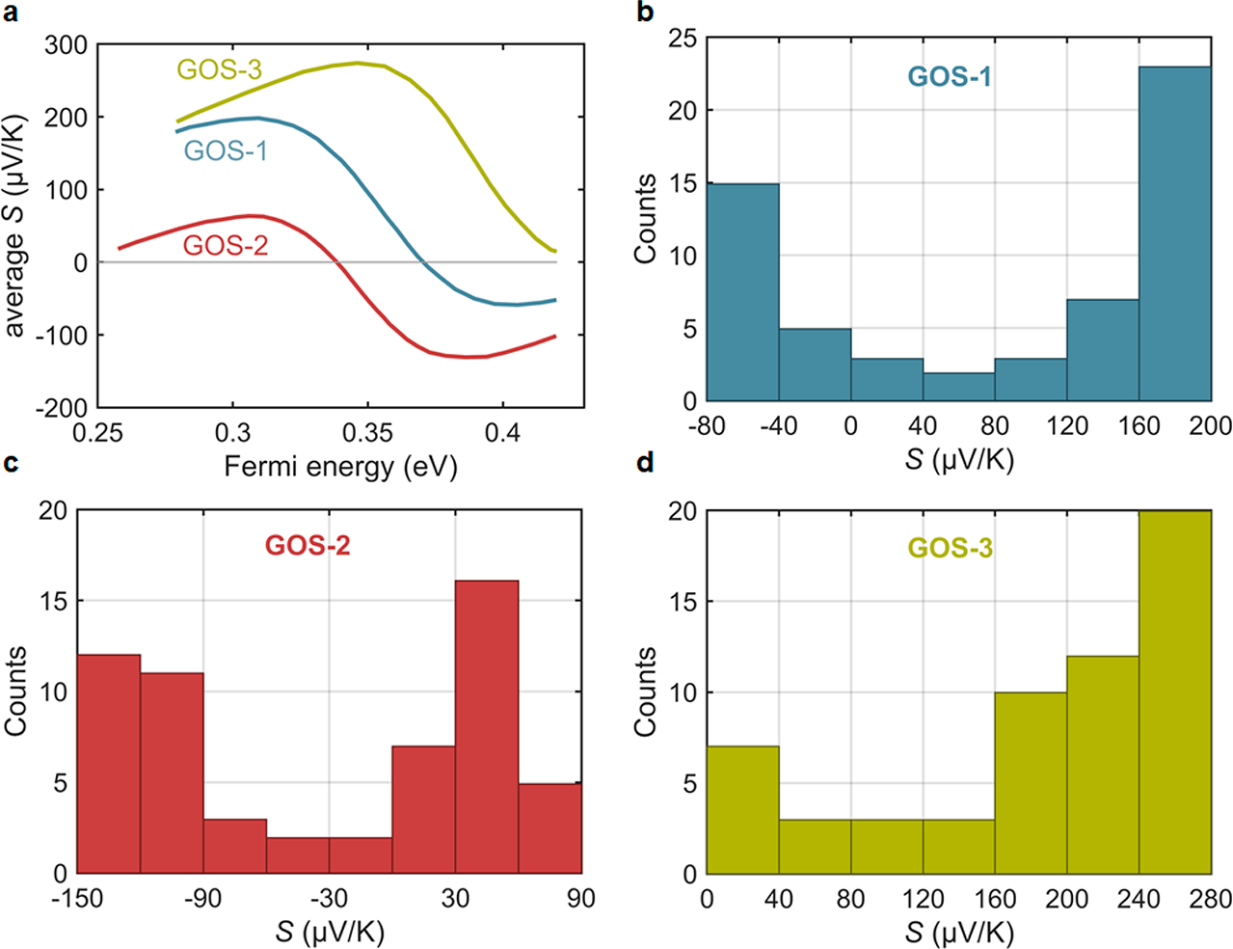
(a) Dependence of the average *S* versus Fermi energy for 20 different tip–C_60_ contact geometries for **GOS-1**, **GOS-2**, and **GOS-3** (in black, red, and green, respectively). (b–d) Histograms of the Seebeck coefficient for these 20 different contact geometries for **GOS-1**, **GOS-2**, and **GOS-3**, respectively. The histograms are obtained by sampling the Seebeck coefficient at different Fermi energies between 0.35 and 0.42 eV, relative to the DFT-predicted Fermi energy *E*_*F*_^*DFT*^ (as shown in Figure S18).

As discussed in the SI, the calculated Seebeck coefficients were found to be sensitive to the location of the metallic tip on the surface of the C_60_ and to the value of the Fermi energy, which can vary across the film due to the presence of adsorbates, such as water. Therefore, values of *S* for a range of tip-C_60_ contact locations and a range of Fermi energies were calculated. These were then used to create the histograms of Seebeck coefficients shown in Figure 5. These are in qualitative agreement with the experimental histograms of Figure 3, with **GOS-3** values of S being positive, whereas **GOS-1** and **GOS-2** histograms possess peaks at both positive and negative values. This suggests that the existence of two peaks in the histograms cannot be considered as an indication of the presence of molecules bound by one or two legs and that the precise shape of Seebeck histograms depends on the range of Fermi energies and tip-C_60_ configurations sampled in the experiments. The sign of the Seebeck coefficient depends on the energetic location of the frontier orbitals relative to the Fermi energy. If the LUMO is closer to the Fermi energy, then the Seebeck coefficient is negative, whereas if the HOMO is closer, the Seebeck coefficient is positive. Bonding of the tip to the unattached phenyl ring was not considered, since the unattached ring points toward the substrate, making it unavailable to the tip.

In the above simulations, each junction contained a single molecule, whereas in the experiments, many molecules are present. The effect of intermolecular interactions in junctions with more than one C_60_ has been considered in a previous work.^33^ This study showed that intermolecular interactions cause small changes in the Seebeck coefficient, but these are negligible compared to the sample-to-sample fluctuations shown in Figures 3 and 5.

In summary, suitably functionalized [60]fullerene derivatives (**1**–**3**) have been covalently linked to 2D graphene either through two *meta*-connected phenyl rings, two *para*-connected phenyl rings, or just a single phenyl ring, resulting in the new systems **GOS1**–**3**. All the [60]fullerene/graphene hybrids have been characterized by spectroscopic and microscopic techniques, revealing the efficient covalent linkage between the two carbon allotropes.

Our work demonstrates that the [60]fullerene derivatives **GOS-1**, **GOS-2**, and **GOS-3** generate exceptionally high quantum thermopowers when covalently anchored to graphene, thus suggesting great promise for molecular/graphene composite materials in thermoelectric applications. The possibility to change the sign of the most probable Seebeck coefficient when utilizing two anchoring groups implies potential for tailoring thermoelectric performance in more complex device architectures in which positive and negative contributions may be combined to build thermoelectric modules.

DFT calculations show that the sign of the Seebeck coefficient of **GOS-1** and **GOS-2** is very sensitive to local variations of the Fermi level, leading to both positive and negative values of the Seebeck coefficient. In contrast, the calculations predict that, for the same local variations in the Fermi energy, the thermopower of **GOS-3** is more robust when anchored to graphene, since its transmission function exhibits a consistently negative slope around the energy range of interest. Fluctuations in energies of the frontier orbitals relative to the Fermi energy are modeled by sampling a range of Fermi energies. The precise range sampled experimentally is unknown, because small changes in the bonding configuration and defects in the graphene will change the Fermi energy relative to frontier orbital energies. Therefore, we sampled a range, which was consistent with experiment. In this sense, although the theory is not predictive, we are able to show that the experiment can be consistent with theory. In conclusion, the geometries and Fermi energies vary so much that a broad distribution of Seebeck coefficients are observed. The sign of the Seebeck coefficient depends on the energetic location of the frontier orbitals relative to the Fermi energy. These are shifted relative to their gas-phase values by the real part of the self-energy, due to contact with the substrate and tip. This shift depends on the strength of the contact, which in turn depends on the orientation of the molecule and details of the linker. Random energy level shifts also occur due to imperfections in the graphene substrate.

## Supporting Information Available

Details about synthesis and further characterization, spectroscopic data, thermogravimetric analysis, transmission electron microscopy, and computational details (PDF)
