# Supplementary material for: Enhanced Thermoelectricity in Metal–[60]Fullerene–Graphene Molecular Junctions
Source: Nano Lett. 2023 Mar 27;23(7):2726–32. doi: 10.1021/acs.nanolett.3c00014 (PMC10103166; doi:10.1021/acs.nanolett.3c00014)
Supplement: Supplementary file 1 — nl3c00014_si_001.pdf [file nl3c00014_si_001.pdf]

Enhanced thermoelectricity in metal-[60]fullerene-graphene molecular junctions

Simon A. Svatek[1,2,§], *Valentina Sacchetti*[1,3], *Laura Rodríguez-Pérez,*[3] *Beatriz M. Illescas,*[3] *Laura Rincón-García*[2], *Gabino Rubio-Bollinger*[2,4], *M. Teresa González*[1], *Steven Bailey*[5], *Colin J. Lambert*[5], *Nazario Martín*[1,3], *Nicolás Agraït*[1,2,4]

[1] Instituto Madrileño de Estudios Avanzados en Nanociencia (IMDEA-Nanociencia), Faraday 9, Ciudad Universitaria de Cantoblanco, 28049, Madrid, Spain

[2] Departamento de Física de la Materia Condensada, Facultad de Ciencias, C/ Francisco Tomás y Valiente 7, Universidad Autónoma de Madrid, 28049, Madrid, Spain

[3] Organic Chemistry Department, Faculty of Chemistry, Universidad Complutense de Madrid, E-28040, Madrid, Spain

[4] Condensed Matter Physics Center (IFIMAC) and Instituto Universitario de Ciencia de Materiales "Nicolás Cabrera" (INC), Facultad de Ciencias, C/ Francisco Tomás y Valiente 7, Universidad Autónoma de Madrid, 28049, Madrid, Spain

[5] Department of Physics, Lancaster University, Lancaster LA1 4YW, UK

[§] Present Address: Universidad Politécnica de Madrid – Instituto de Energía Solar, Avenida Complutense 30, 28040 Madrid, Spain

Corresponding Authors

*** e-mail: c.lambert@lancaster.ac.uk, nicolas.agrait@uam.es, nazmar@ucm.es**

S1. Synthesis and characterization

General. Reagents and solvents were purchased as reagent grade and used without further purification. CVD-graphene on 300 nm SiO_2/Si wafers (GOS) was purchased from Graphenea (www.graphenea.com). Compounds **1** and **3** were prepared according to previously reported procedures.[1] For column chromatography, silica gel 60 (230-400 mesh, 0.040-0.063 mm) was purchased from E. Merck. Thin Layer Chromatography (TLC) was performed on aluminum sheets coated with silica gel 60 F_{254} purchased from E. Merck, visualization by UV light. IR spectra (cm^{-1}) were measured on an ATI Mattson Genesis Series FTIR instrument. NMR spectra were recorded on a Bruker AC 300, AC 500 or AC 700 with solvent peaks as reference. MALDI-TOF-mass spectra were carried out on a Bruker BIFLEXTM matrix-assisted laser desorption time-of-flight mass spectrometer using 2-[(*E*)-3-(4-*tert*-butylphenyl)-2-methylprop-2-enylidene]-propanedinitrile (DCTB) as matrix. Electrospray ionization (ESI) mass spectra were recorded with an Esquire 6000 ESI-Ion Trap from Bruker Daltonics using CH_2Cl_2/MeOH as solvent system.

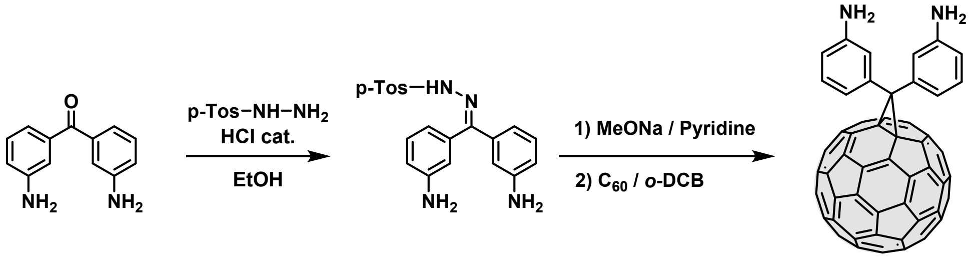

4 5 2

Scheme S1. Synthesis of compound **2**.

Synthesis of 3,3′-diaminobenzophenone tosylhydrazone (5)

Under argon atmosphere, a solution of 3,3′-diaminobenzophenone **4** (808 mg, 3.81 mmol) and *p*-tosylhydrazide (710 mg, 3.81 mmol) in 50 mL of toluene and 7 mL of ethanol was refluxed for 24 h. After cooling to room temperature, the solvent was evaporated, and the crude purified by column chromatography with hexane/AcOEt (1:1) to achieve a brown solid. Yield: 79%; FTIR (KBr) υ:3451, 3383, 2924, 2854, 1924, 1624, 1489, 1452, 1381, 1324, 1240, 1165, 1091, 1051, 1016, 900, 790, 701, 670, 582, 548 cm^{-1}; ^{1}H NMR (300 MHz, MeOH): δ 7.84 (d, *J* = 8.2 Hz, 2H), 7.41 (d, *J* = 8.0 Hz, 2H), 7.23 (t, *J* = 7.8 Hz, 1H), 7.01 (t, *J* = 7.7 Hz, 1H), 6.81 (d, *J* = 8.1 Hz, 2H), 6.70 (d, *J* = 7.9 Hz, 2H), 6.46 (s, 1H), 6.39 (d, *J* = 7.5 Hz, 1H), 2.44 (s, 3H); ^{13}C NMR (125 MHz, MeOD): δ 159.75, 147.27, 141.65, 140.93, 139.97, 138.16, 129.30, 128.99, 128.27, 118.65, 117.82, 115.23, 21.52. MALDI-TOF: *m/z* theoretical for $C_{77}H_{25}NO_5$ 380.13, exp M^+ 380.83.

Synthesis of compound 2

To a solution of 3,3'-diaminobenzophenone tosylhydrazone **5** (155 mg, 0.41 mmol) in 8 mL of dry pyridine, sodium methoxyde (22 mg, 0.41 mmol) was added under argon atmosphere. After stirring at room temperature for 15 minutes, a solution of [60]fullerene (211 mg, 0.29 mmol) in 20 mL of *o*-DCB was added at once and the mixture was heated at 180°C for 18 h. After cooling to room temperature, the solvent was evaporated and the remaining solid purified by column chromatography on SiO_2 (CS_2; Tol/AcOEt 8/2). Yield: 30%; FTIR (KBr) υ: 3451, 3385, 2923, 2854, 1611, 1489, 1456, 1382, 1312, 1277, 1162, 862, 771, 697, 569, 525 cm^{-1}; ^{1}H NMR (300 MHz, DMSO): δ 7.29 (dd, *J* = 1.9 Hz, 4H), 7.14 (t, *J* = 8.0 Hz, 2H), 6.57 (d, *J* = 8.6 Hz, 2H), 5.31 (s, 4H); ^{13}C NMR (175 MHz, DMSO): δ 149.30, 148.64, 145.35, 144.64, 144.53, 144.32, 144.16, 144.01, 143.65, 143.38, 142.40, 142.37, 142.12, 141.84, 141.63, 140.17, 139.54, 137.42, 129.17, 128.25, 118.63, 116.30, 113.64, 79.69, 59.56, 39.52; MALDI-TOF: *m/z* theoretical for $C_{77}H_{25}NO_5$ 916.10, exp M^+ 916.16.

General Procedure for the Functionalization of FLG

Exfoliated graphene suspensions in *o*-DCB were utilized as produced for further covalent modification. 50 mL of graphene solution were reacted with a 5 mL solution of the corresponding methanofullerene **1**, **2** or **3** (0.065 mmol) in *o*-DCB. The solution was sonicated while deoxigenating with argon for 90 minutes. Isoamyl nitrite (0.26 mmol) was slowly added to the reaction mixture and heated at 70 °C for 24 h under inert atmosphere. The final product was washed in a 0.1 µm size PTFE membrane several times with o-DCB, CH_2Cl_2 and MeOH.

FLG-1: IR (KBr): ν = 2928, 2857, 1655, 1579, 1444, 1402, 1157, 1068, 756, 525 cm^{-1}; TGA: weight loss and temperature desorption (organic anchoring groups): 24.82 %, 650 °C; Raman: I_D/I_G = 0.14; XPS: % atomic: C (284.6 eV) = 87.2, O (531.6 eV) = 12.1, N (399.6 eV) = 0.65.

FLG-2: IR (KBr): ν = 2928, 2857, 1653, 1579, 1454, 1401, 1038, 784, 525 cm^{-1}; TGA: weight loss and temperature desorption (organic anchoring groups): 34.70 %, 650 °C; Raman: I_D/I_G = 0.29; XPS: % atomic: C (284.6 eV) = 80.21, O (532.6 eV) = 19.1, N (399.6 eV) = 0.7.

General Procedure for the Functionalization of GOS

A solution of the corresponding diphenylmethanofullerene **1**, **2** or **3** (0.008 M) in chlorobenzene and CS_2 was diluted to 10^{-4}M in chlorobenzene and deoxygenated for 1 h. Isoamyl nitrite (0.0004 mmol) was slowly added to the reaction mixture that was subsequently drop casted (one drop) onto the GOS. The material was kept at room temperature under inert atmosphere for 4 h and subsequently washed several times with *o*-DCB, CH_2Cl_2 and MeOH. **GOS-1**: Raman: I_D/I_G = 0.26; **GOS-2**: Raman: I_D/I_G = 0.54; **GOS-3**: Raman: I_D/I_G = 0.23

Raman spectroscopy

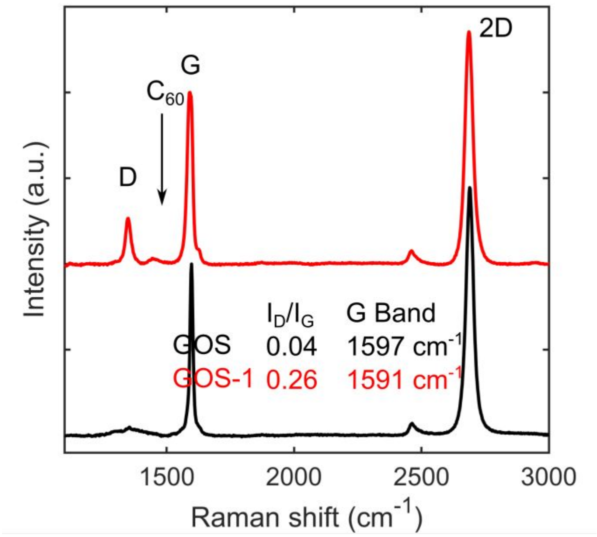

Figure S1: Raman spectra of pristine **GOS** (black), and **GOS-1** (red) under 532 nm laser excitation wavelength.

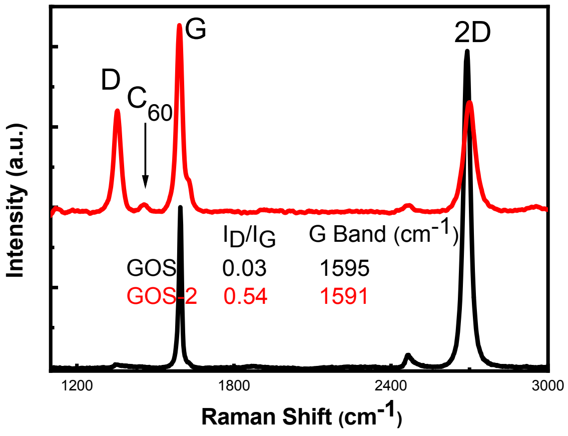

Figure S2: Raman spectra of pristine **GOS** (black), and **GOS-2** (red) under 532 nm laser excitation wavelength.

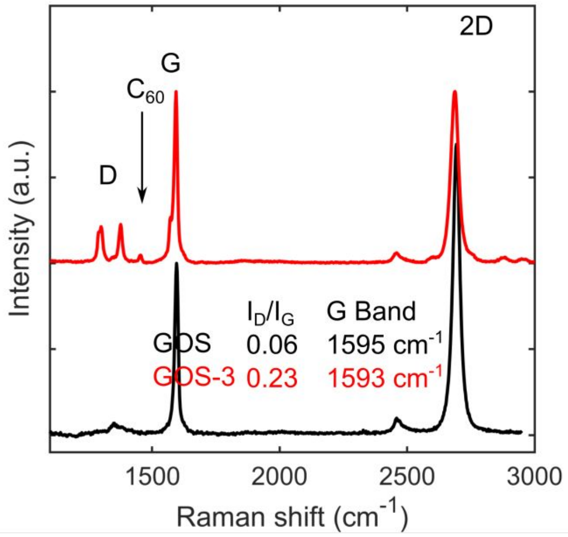

Figure S3. Raman spectra of pristine **GOS** (black), and **GOS-3** (red) under 532 nm laser excitation wavelength.

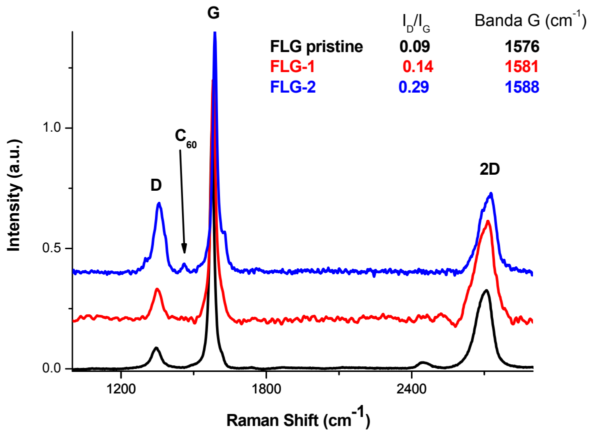

Figure S4. Raman spectra of pristine **FLG** (black), **FLG-1** (red) and **FLG-2** (blue) under 532 nm laser excitation wavelength.

TGA

Thermogravimetric analysis (TGA) under inert atmosphere was carried out on the FLG derivatives to estimate the degree of molecules attached onto their surface (Figure S4). Pristine FLG shows high thermal stability until 800°C degrees with a slight weight loss due to some physisorbed solvent molecules used in the exfoliation process. For both **FLG-C_{60}** covalent compounds, two weight loss processes were observed. The first weight loss step can be assigned to the thermal detaching of the C_{60} moiety followed by the second weight loss where the C_{60} is decomposed. The total weight loss for **FLG-2** (34.70 %) is significantly higher compared to **FLG-1** (24.80 %) which can be traduced in a higher degree of functionalization. The average molar content can be estimated for **FLG-1** in 1 organic molecule per 224 carbon atoms and for **FLG-2** in 1 organic molecule per 139 carbon atoms.

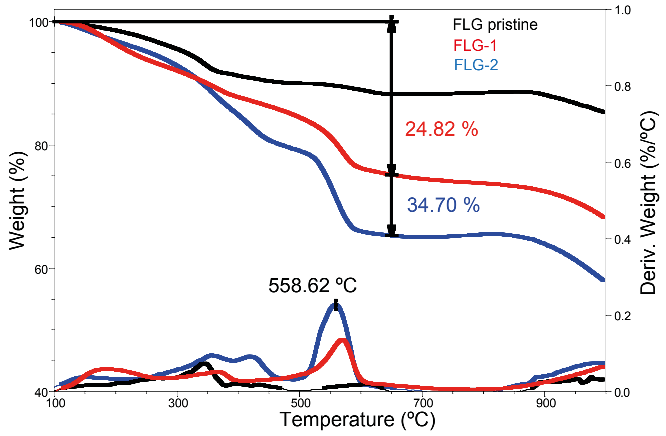

Figure S5. TGA analysis under inert conditions of exfoliated FLG (black), and the nanoconjugates **FLG-1** (red), **FLG-2** (blue).

FTIR

Fullerene derivatives **1** or **2** were identified anchored on the FLG skeleton by Fourier Transform infrared spectroscopy (FTIR). Figure S5 shows the IR spectroscopy of **FLG-1** and **FLG-2** compared to pristine FLG. Among the characteristic peaks are the skeletal in plane vibrations of graphene at 1581 cm^{-1} as well as some vibrational peaks at 2928 and 2857 cm^{-1}, that could be related to aliphatic carbon atoms. But more important is the characteristic vibrational peaks of pristine C_{60} that are shown around 1454, and 525 cm^{-1} for all FLG final conjugates. These peaks are also present in the organic molecules **1** and **2**.

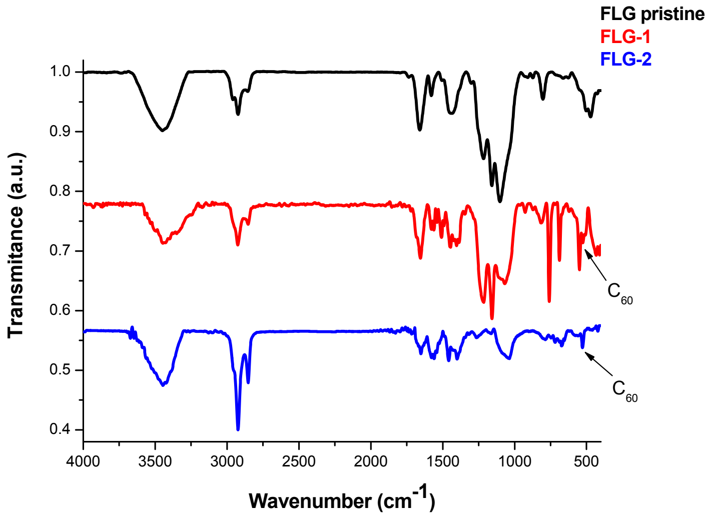

Figure S6. FTIR spectra of **FLG-1** (red) and **FLG-2** (blue) compared to exfoliated **FLG**.

XPS analysis

As further evidence, XPS analysis was recorded for **FLG-1** and **FLG-2**. The survey spectra of **FLG-1** and **FLG-2** exhibit a main C 1s component together with two more signals assigned to O 1s and N 1s (Figures S6 and S7). The 12 % oxygen contribution observed for **FLG-1** is the same obtained for pristine FLG, indicating that no extra oxygen atoms are covalently anchored on the FLG surface during the radical addition reaction with **1**. However, for both **FLG-1** and **FLG-2** the N 1s peak could be related to unreacted amine groups that are still present in the organic molecule, being the high-resolution spectra of N 1s made up of only one component. The high-resolution C 1s core level spectra of both materials **FLG-1** and **FLG-2** present, besides the four expected components (photoelectrons emitted from sp^2 carbon atoms, sp^3 carbon atoms, oxidized carbon atoms in the C–O bonds and the π–π* shake up structure of graphene sheets), two new contributions that could be assigned to C 1s shake-up satellite peaks of C_{60}.[2,3]

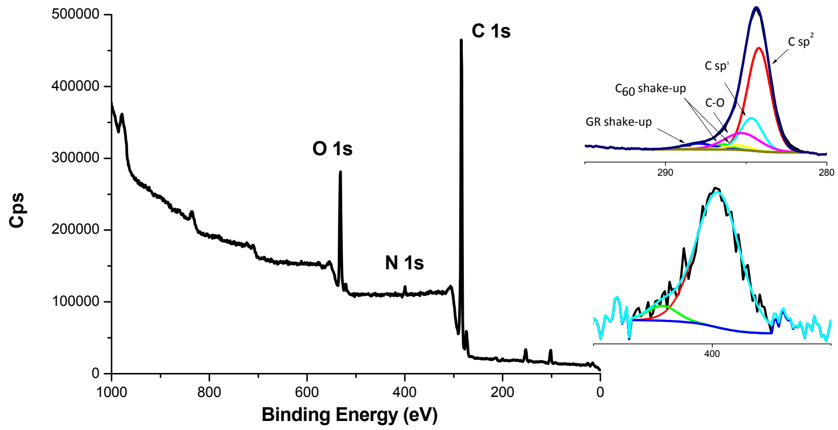

Figure S7. XPS survey spectra of **FLG-1** with inset of the high-resolution spectra of C 1s (up) and N 1s (down).

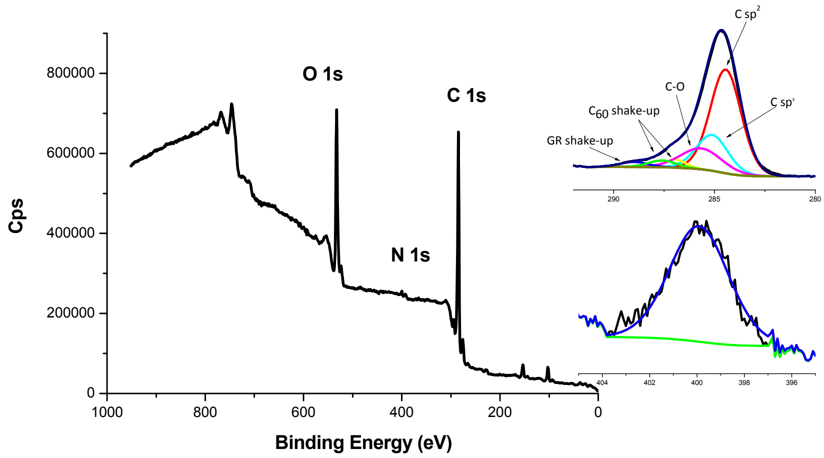

Figure S8. XPS survey spectra of **FLG-2** with inset of the high-resolution spectra of C 1s (up) and N 1s (down).

TEM

Transmission electron microscopy (TEM) images of **FLG-1** and **FLG-2** are illustrated in Figure S8. Interestingly, some spherical species with diameter of ~1nm are clearly observed at the edges of the functionalized material. These spherical species are not observable in pristine FLG TEM micrographs under identical conditions and could be attributed to the covalently attached organic molecules based on C_{60}, since its diameter is comparable with the diameter reported for C_{60}.[4,5]

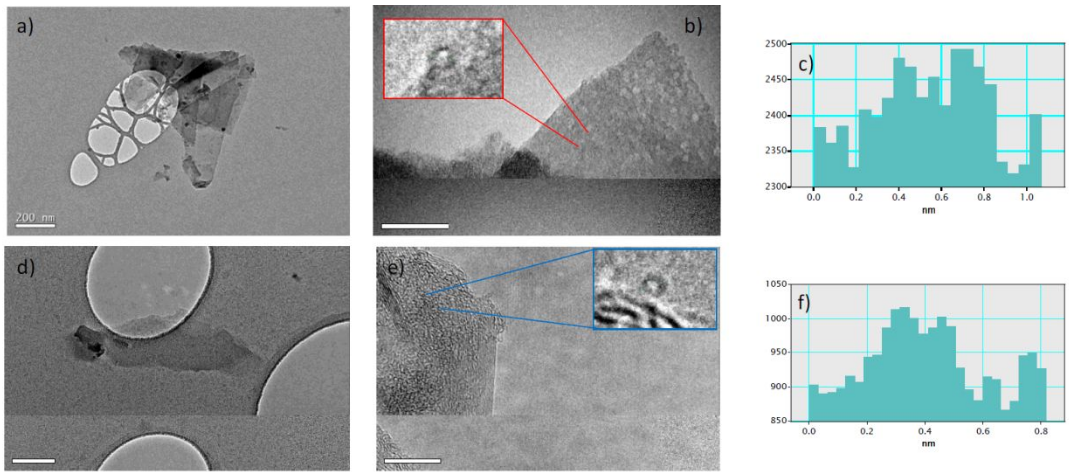

Figure S9: TEM micrographs of **FLG-1** with scale bar 200 nm (a), 20 nm (b) and representative image of the width profile of the fullerene attached to the aggregate (c). TEM micrographs of **FLG-2** with scale bar 100 nm (d), 10 nm (e) and representative image of the width profile of the fullerene attached to the aggregate (f).

S2. Computational details

Density Functional Theory (DFT) was used to investigate transport through a graphene monolayer functionalized by three derivatives a) **GOS-1** *para*-connected, b) **GOS-2** *meta*-connected and c) **GOS-3** single *para*-connected diphenylmethanofullerene. In each case the aryl radicals are allowed to bond with a graphene surface. Experimental c-AFM measurements demonstrate that the sign of the Seebeck coefficient (thermopower) can be positive or negative in both dibenzyl derivatives, whereas the single benzyl derivative is positive. The aim of these calculations is to help understand the change in sign and magnitude of the Seebeck coefficient observed as a function of different bonding geometries between C_{60}-dibenzyl and a graphene sheet substrate.

The energetics and resulting conformations of a C_{60}-dibenzyl unit bonded to a graphene surface via activated aryl radicals were obtained from the SIESTA[6] implementation of density functional theory combined with the van der Waals density functional,[7,8] which uses the revPBE35 revised version of Perdew, Burke and Ernzerhof generalized-gradient approximation exchange correlational functional.[9] The van der Waals functional used in SIESTA is a universal non-local density functional applicable to arbitrary geometries.[10] To ensure accuracy, the inter atomic forces were relaxed to optimize the geometry to a value less than 20 meV/Å using extended

double zeta polarized basis sets of pseudo atomic orbitals. In calculating the binding energies, basis set superposition errors were minimized by retaining ghost states, as prescribed in the counterpoise method.[11] Once the conformer geometries shown in Figure S9 were optimized, the mean-field Hamiltonian and overlap matrices were used to calculate the electronic and thermoelectronic properties from our transport code GOLLUM.[12]

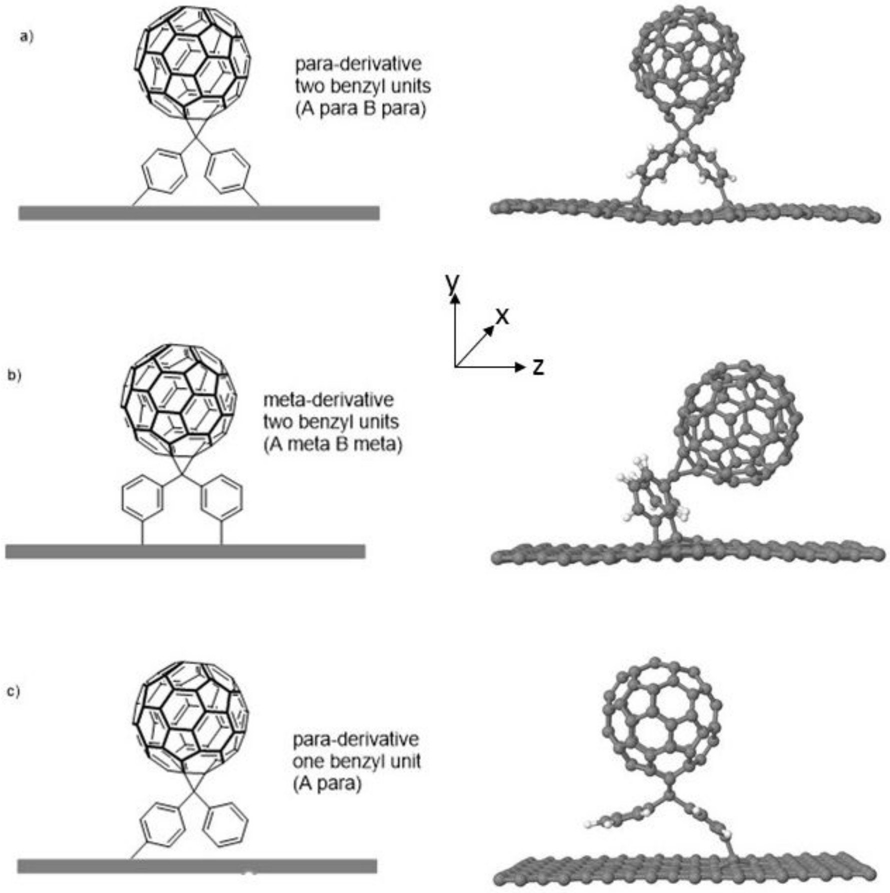

Figure S10: Simulated structures a) **GOS-1**, b) **GOS-2** and c) **GOS-3** on the left and the relaxed geometries from the theoretical calculations on the right. The graphene sheet with 216 carbon atoms is periodic in both the in-plane directions.

The three energetically relaxed forms of covalently bonded C_{60}-diphenyl to graphene are shown in Figure S10, where in a) both phenyl units form covalent bonds to the graphene in a para configuration, while in b) both phenyl units are meta-covalently bonded to the graphene and in c) one phenyl unit is para-covalently bonded to the graphene.

The calculations leading to the successful SIESTA simulation of the Tour reaction involve carefully placing the reactive aryl units close to the pristine graphene surface. This process is illustrated in Figure S11, where the right-hand panel shows time frames of the conjugate gradient relaxation process in SIESTA for dibenzyl units of type a), where to save computation time the C_{60} is removed (and replaced by two hydrogen atoms) and the graphene sheet is reduced to 96

atoms. It was found that randomly distributing the initial starting configurations of the dibenzyl units of structures a) and b) over the graphene surface resulted in no covalent bonding in over 60% of the simulations. Nevertheless, when the reactive carbon atoms in the two dibenzyl units for structure a) are then located as shown in the left-hand image of Figure S11 in close proximity to the graphene surface, frame 2 shows that the dibenzyl unit can detach from the surface, but then recombine, as shown in frames 3-5. Similarly for structures b), carefully locating the reactive carbon atoms on the graphene surface assures covalent bonding. For structure c) the single para derivative with one benzyl unit again has approximately a 60% probability to detach, even when the reactive carbon atom is located close to a selected atom on the graphene surface. Once the relaxed configurations of covalently bonded structures a), b) and c) have been found, the C_{60} is reattached, and the graphene sheet is roughly doubled in size to contain 216 carbon atoms. The system is again relaxed using the bonded conformations. To model the junction the top contact is chosen to be gold, as an archetypal metallic electrode, and the most energetically favorable tip to C_{60}-dibenzyl geometry is found. This is shown in Figure S12, where the Au-C bond is shown to form two legs of length d = 2.5 Å placed equidistantly between two carbon atoms on the C_{60} forming the edge of a pentagonal ring.

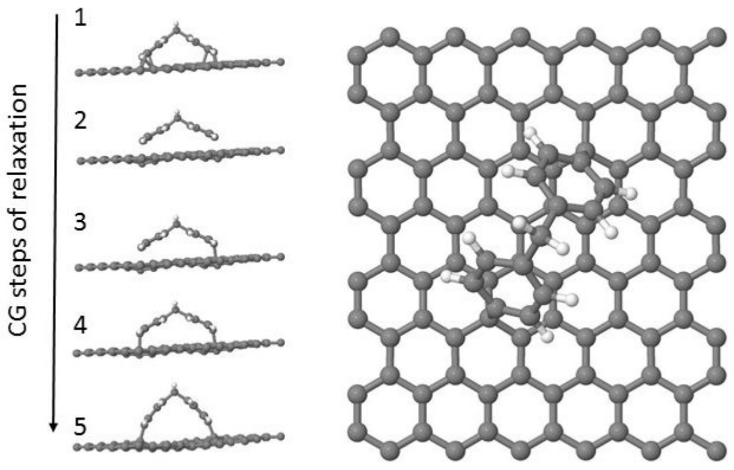

Figure S11: Time frames (1-5) of the conjugate gradient steps for the relaxation of structure a), where the aryl units attach to the graphene surface in the left-hand image at the positions of the active aryl site shown in the right-hand image. The C_{60} molecule has been replaced by two hydrogen atoms to save computational time.

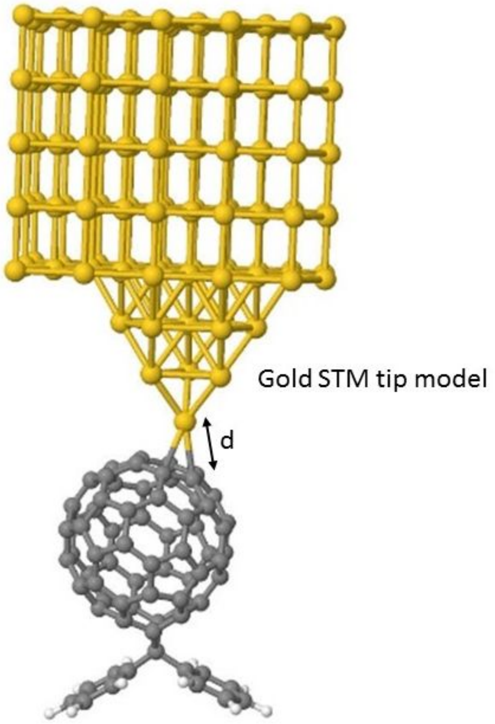

Figure S12: The most energetically favourable geometry to for the STM metallic tip and C_{60}. The two bonds shown between the tip and C_{60} surface are of length *d* = 2.5 Å and form an equilateral triangle with the two carbon atoms forming an edge of a pentagon.

Finally, the relaxed geometry for systems a) **GOS-1**, b) **GOS-2** and c) **GOS-3** are shown in Figure S10 and the structures with the STM tip attached are shown in Figure S13. SIESTA then calculates the mean-field Hamiltonian of these relaxed structures without further conjugate gradient relaxation.

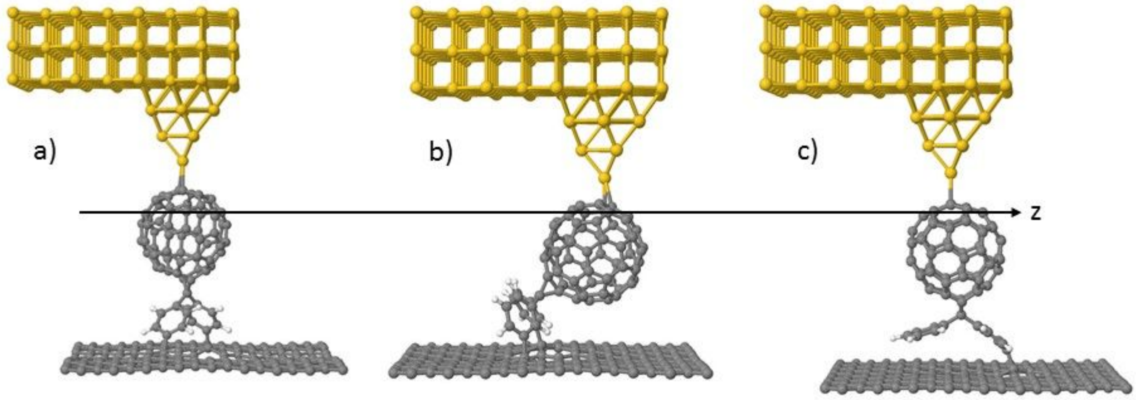

Figure S13: Modelled c-AFM experimental setup to measure the electronic and thermoelectric properties for the meta and para covalent bonding of C_{60}-diphenyl to a graphene sheet.

The mean-field Hamiltonian and overlap matrices are then used by the transport code GOLLUM to calculate the transmission coefficients $T(E)$ from which the thermoelectric properties are obtained.

The Seebeck coefficient (or thermopower) is defined by *S* = -*ΔV/ΔT* and provides a measure of a voltage difference *ΔV* created by temperature difference *ΔT*. The Seebeck coefficient at a temperature *T* is calculated from

$$S(T) = -\frac{L_1}{|e|TL_0} \tag{1}$$

where e is the charge of an electron and the coefficients L_n are given by

$$L_n(T) = \int_{-\infty}^{+\infty} dE(E - E_F)^n T(E)(-\frac{\partial f(E)}{\partial E}) \tag{2}$$

In the above expression, $f(E) = (1 + \exp{(E - E_F/k_BT)})^{-1}$is the Fermi-Dirac probability distribution function with E_F the Fermi energy and $T(E)$ is the transmission coefficient. If the transmission coefficient changes only slowly on the scale of k_BT, then the sign and magnitude of the Seebeck coefficient are given by the negative of the slope of log $T(E)$ at the Fermi energy. For each of the three molecules **GOS-1**, **GOS-2** and **GOS-3**, Figure S14, shows *T(E)* plotted on a linear scale, while Figure S15 shows a plot of $\log_{10} T(E)$ vs. *E*.

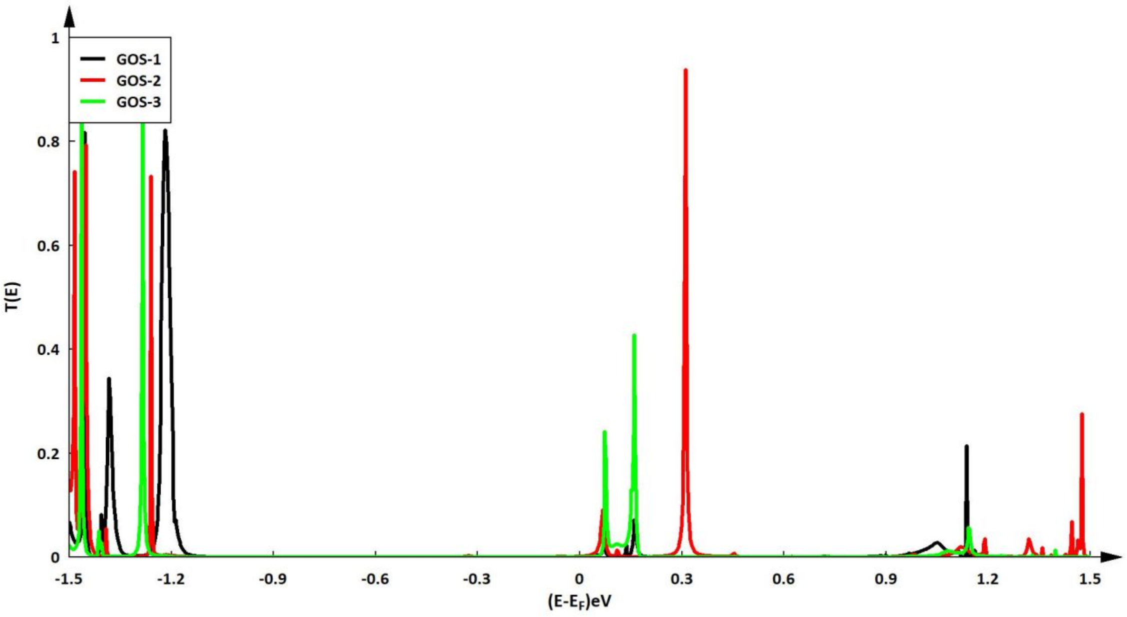

Figure S14. Transmission functions *T(E)* for **GOS-1**, **GOS-2** and **GOS-3** in an energy range which captures the LUMO+1 and HOMO-1 levels.

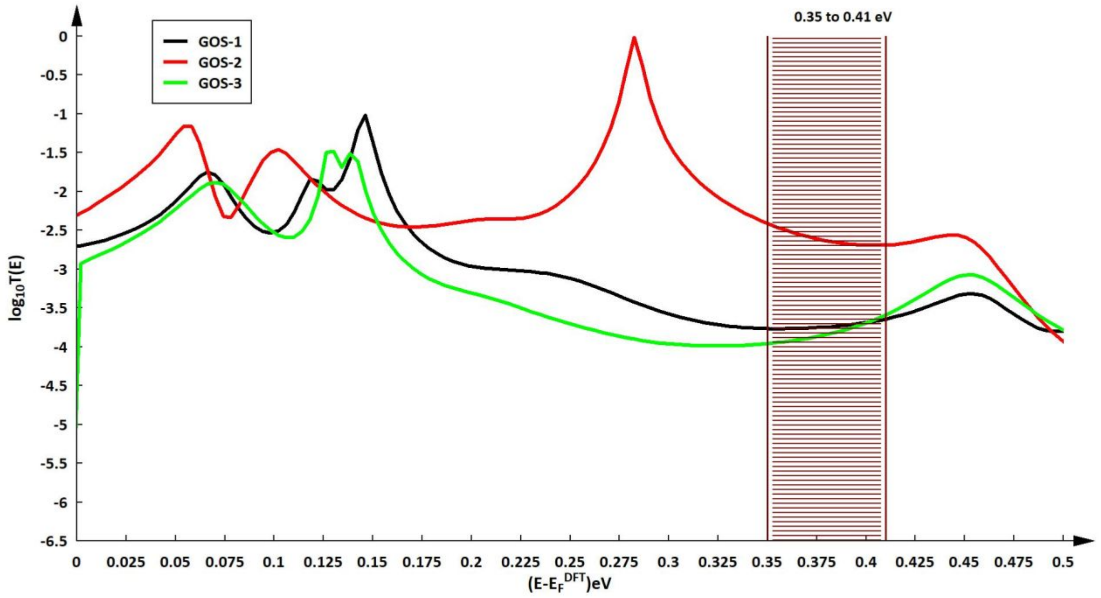

Figure S15. A plot of the transmission coefficient of Figure S14 on a logarithmic scale. The shaded energy range coincides with the tail of the LUMO levels.

Substituting the above results for *T(E)* into equation (1) yields the Seebeck coefficient for each molecule. Since the value of the Seebeck coefficient depends on the Fermi energy E_F, Figure S16 shows *S* (μV/K) as a function of the Fermi energy for a range between -0.5 and 0.5 eV, relative to the DFT-predicted value.

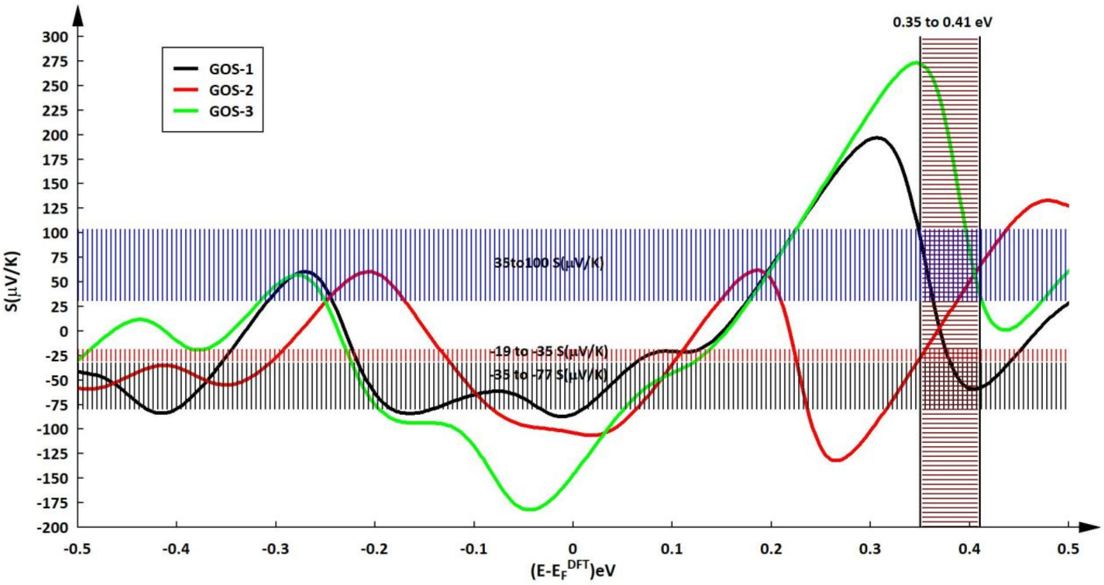

Figure S16. Seebeck coefficient as a function of the Fermi energy at 300 K for the three molecules **GOS-1**, **GOS-2** and **GOS-3**.

The Fermi energy range relevant experimentally can depend on environmental factors, such as the presence of adsorbed water. Therefore, we ask if there is a single Fermi energy range which reproduces the experimentally measured trends in the Seebeck coefficients of all three molecules. The more detailed plot in Figure S17 shows that this is indeed the case, provide the experimental Fermi energy lies in the range 0.35 to 0.41 eV relative to the DFT-predicted value, since in this range the Seebeck coefficients of **GOS-3** are consistently positive, whereas those of **GOS-1** and **GOS-2** take both positive and negative values.

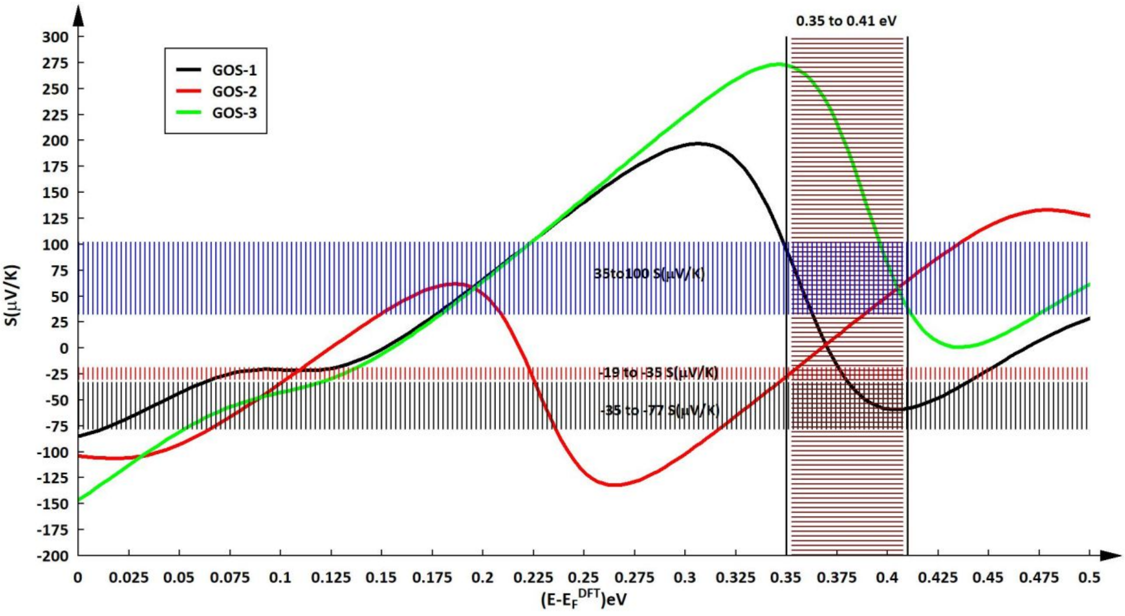

Figure S17. Seebeck coefficient for the optimized contact geometries for **GOS-1**, **GOS-2** and **GOS-3** for Fermi energies between 0.0 and 0.5 relative to the DFT-predicted value E_F^{DFT}.

The experimental histograms are the result of many contact geometries between the tip and C_{60} and also tip-C_{60} distance *d,* which may vary relative to the optimized value of *d* = 2.5 Å. Therefore, to model this behavior, the tip was placed at twenty locations perpendicular to the cap of the C_{60}, each at the optimized distance of *d* = 2.5 Å, as illustrated in Figure S18. In addition, two slightly stretched or slightly compressed values of the optimized distance *d* were considered.

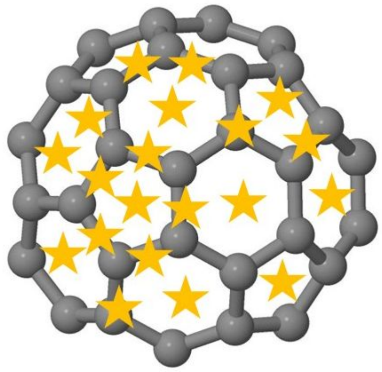

Figure S18. 20 different tip locations on the fullerene surface.

In summary, we used a randomly distribution of initial starting configurations in over the graphene surface. These resulted in no covalent bonding in over 60% of the simulations. Those that did bond led to the same relaxed structures shown in Figure S10. Starting from these, 20 tip-sample geometries were the considered, as shown in Figure S18. The different geometries gave a series of transmission curves, from which Seebeck coefficients were evaluated at discrete values of the Fermi energy in the shaded range shown in Figure S17. These values were then used to construct the histograms of *S* for each molecule, shown in Figure 4 of the main text. The resulting distributions of Seebeck coefficients are in qualitative agreement with the experimental results shown in Figure 2 of the main text and suggest that the measured fluctuations in *S* arise from a combination of variations in the tip-C_{60} contact and local variations in energies of frontier orbitals relative to the Fermi energy.

$\mathbf{C1}s$ shakeup spectrum of $\mathbf{C60}$: Global charge-transfer satellites and their relation to the x-ray threshold singularities in macroscopic systems

References

1. Gómez R.; Segura J. L.; Martín N. Highly Efficient Light-Harvesting Organofullerenes. *Org. Lett.* **2005**, *7*, 717-720.
2. Guan J.; Chen X.; Wei T.; Liu F.; Wang S.; Yang Q.; Lu Y.; Yang S. Directly bonded hybrid of graphene nanoplatelets and fullerene: facile solid-state mechanochemical synthesis and application as carbon-based electrocatalyst for oxygen reduction reaction. *J. Mater. Chem. A.* **2015**, *3*, 4139-4146.
3. Enkvist C.; Lunell S.; Sjögren B.; Svensson S.; Brühwiler P. A.; Nilsson A.; Maxwell A. J.; Mårtensson N. C_{1s} shakeup spectrum of C_{60}: Global charge-transfer satellites and their relation to the x-ray threshold singularities in macroscopic systems. *Phys. Rev. B.* **1993**, *48*, 14629-14637.
4. Zhang X.; Liu Z.; Huang Y.; Wan X.; Tian J.; Ma Y.; Chen Y. Synthesis, Characterization and Nonlinear Optical Property of Graphene-C_{60} Hybrid. *J. Nanosci. Nanotechnol.* **2009**, *9*, 5752-5756.
5. Zhang Y.; Ren L.; Wang S.; Marathe A.; Chaudhuri J.; Li G. Functionalization of graphene sheets through fullerene attachment. *J. Mater. Chem.* **2011**, *21*, 5386-5391.
6. Soler J. M.; Artacho E.; Gale J. D.; García A.; Junquera J.; Ordejón P.; Sánchez-Portal D. The SIESTA method forab initioorder-Nmaterials simulation. *J. Phys. Condens. Matter* **2002**, *14*, 2745-2779.
7. Dion M.; Rydberg H.; Schröder E.; Langreth D. C.; Lundqvist B. I. Van der Waals Density Functional for General Geometries. *Phys. Rev. Lett.* **2004**, *92*, 246401.
8. Langreth D. C.; Dion M.; Rydberg H.; Schröder E.; Hyldgaard P.; Lundqvist B. I. Van der Waals density functional theory with applications. *Int. J. Quantum Chem.* **2005**, *101*, 599-610.
9. Zhang Y.; Yang W. Comment on ``Generalized Gradient Approximation Made Simple''. *Phys. Rev. Lett.* **1998**, *80*, 890-890.
10. Jansen H. B.; Ros P. Non-empirical molecular orbital calculations on the protonation of carbon monoxide. *Chem. Phys. Lett.* **1969**, *3*, 140-143.
11. Boys S. F.; Bernardi F. The calculation of small molecular interactions by the differences of separate total energies. Some procedures with reduced errors. *Mol. Phys.* **1970**, *19*, 553-566.
12. Ferrer J.; Lambert C. J.; García-Suárez V. M.; Manrique D. Z.; Visontai D.; Oroszlany L.; Rodríguez-Ferradás R.; Grace I.; Bailey S. W. D.; Gillemot K.; Sadeghi H.; Algharagholy L. A. GOLLUM: a next-generation simulation tool for electron, thermal and spin transport. *New J. Phys.* **2014**, *16*, 093029.
